# Assessment of Sodium MRI at 7 Tesla as Predictor of Therapy Response and Survival in Glioblastoma Patients

**DOI:** 10.3389/fnins.2021.782516

**Published:** 2021-12-01

**Authors:** Daniel Paech, Sebastian Regnery, Tanja Platt, Nicolas G. R. Behl, Nina Weckesser, Paul Windisch, Katerina Deike-Hofmann, Wolfgang Wick, Martin Bendszus, Stefan Rieken, Laila König, Mark E. Ladd, Heinz-Peter Schlemmer, Jürgen Debus, Sebastian Adeberg

**Affiliations:** ^1^Division of Radiology, German Cancer Research Center (DKFZ), Heidelberg, Germany; ^2^Department of Radiation Oncology, Heidelberg University Hospital, Heidelberg, Germany; ^3^Clinical Cooperation Unit Radiation Oncology, German Cancer Research Center (DKFZ), Heidelberg, Germany; ^4^Division of Medical Physics in Radiology, German Cancer Research Center (DKFZ), Heidelberg, Germany; ^5^Siemens Healthcare GmbH, Erlangen, Germany; ^6^Department of Neurology, Heidelberg University Hospital, Heidelberg, Germany; ^7^Department of Neuroradiology, Heidelberg University Hospital, Heidelberg, Germany; ^8^Faculty of Physics and Astronomy and Faculty of Medicine, University of Heidelberg, Heidelberg, Germany

**Keywords:** brain neoplasm/secondary cases, glioblastoma, response assessment, magnetic resonance imaging, ultra-high field (7 tesla), sodium MRI

## Abstract

The purpose of this work was to prospectively investigate sodium (^23^Na) MRI at 7 Tesla (T) as predictor of therapy response and survival in patients with glioblastoma (GBM). Thus, 20 GBM patients underwent ^23^Na MRI at 7T before, immediately after and 6 weeks after chemoradiotherapy (CRT). The median tissue sodium concentration (TSC) inside the whole tumor excluding necrosis was determined. Initial response to CRT was assessed employing the updated response assessment in neuro-oncology working group (RANO) criteria. Clinical parameters, baseline TSC and longitudinal TSC differences were compared between patients with initial progressive disease (PD) and patients with initial stable disease (SD) using Fisher’s exact tests and Mann-Whitney-U-tests. Univariate proportional hazard models for progression free survival (PFS) and overall survival (OS) were calculated using clinical parameters and TSC metrics as predictor variables. The analyses demonstrated that TSC developed heterogeneously over all patients following CRT. None of the TSC metrics differed significantly between cases of initial SD and initial PD. Furthermore, TSC metrics did not yield a significant association with PFS or OS. Conversely, the initial response according to the RANO criteria could significantly predict PFS [univariate HR (95%CI) = 0.02 (0.0001–0.21), *p* < 0.001] and OS [univariate HR = 0.17 (0.04–0.65), *p* = 0.005]. In conclusion, TSC showed treatment-related changes in GBM following CRT, but did not significantly correlate with the initial response according to the RANO criteria, PFS or OS. In contrast, the initial response according to the RANO criteria was a significant predictor of PFS and OS. Future investigations need to elucidate the reasons for treatment-related changes in TSC and their clinical value for response prediction in glioblastoma patients receiving CRT.

## Introduction

The overall prognosis of glioblastoma (GBM) remains poor despite gross tumor resection followed by chemoradiotherapy (CRT), but long-term survival can be achieved in a small subgroup of patients (Stupp et al., 2005, 2009). In this context, the early identification of tumor relapse remains a major challenge. Current assessment of response to therapy is based on the updated response assessment in neuro-oncology working group (RANO) criteria, which classify clinical follow-up MRI (Wen et al., 2010; Johnson et al., 2019). This approach suffers essential limitations due to the incidence of pseudo-progressions (PsPD) especially shortly after treatment (de Wit et al., 2004; Wen et al., 2010). Repeated imaging studies are necessary to rule out PsPD (Wen et al., 2010; Johnson et al., 2019), which impedes early identification of tumor relapse and delays potential 2nd-line therapies as part of personalized treatment approaches. Consequently, imaging techniques that enable an early or even real-time response assessment to guide therapy protocols are highly desirable.

Sodium (^23^Na) MRI has been used within animal experiments (Schepkin et al., 2005, 2006) and mostly case reports (Thulborn et al., 2009; Laymon et al., 2012; Haneder et al., 2015) to monitor treatment associated changes in GBM consecutive to CRT. Here, ^23^Na MRI demonstrated promising features as it seems to offer complementary information to routine imaging modalities (Laymon et al., 2012; Haneder et al., 2015; Regnery and Platt, 2021). Quite recently, an investigation of ^23^Na MRI at 3 Tesla (T) in GBM patients suggested that the development of tissue sodium concentration (TSC) during CRT might reflect tumor cell kill (Thulborn et al., 2019). Thus, ^23^Na MRI is a promising functional imaging technique in neuro-oncology (Regnery and Platt, 2021). However, ^23^Na MRI is limited by a comparatively low signal-to-noise ratio (SNR) because ^23^Na-ions are far less abundant than the protons used for conventional MRI techniques (Shah et al., 2016). Here, the advent of ultra-high field (UHF) MRI techniques opens new perspectives in ^23^Na imaging by providing increased SNR (Konstandin and Schad, 2014; Shah et al., 2016). This longitudinal prospective study employed a 7T MRI scanner to investigate the development of TSC in 20 GBM patients following CRT. Based on previous reports, we explored the capacity of TSC to reflect therapy response and therefore predict progression free survival (PFS) as well as overall survival (OS).

## Patients and Methods

### Patients

20 GBM patients prospectively underwent ^23^Na imaging on a 7 Tesla MRI system before (t_0_), immediately after (t_1_) and 6 weeks after (t_2_) (C)RT. Inclusion criteria were an age ≥ 18 years, histological proof of GBM, residual disease on clinical imaging after potential tumor resection, currently planned (C)RT and the absence of ferromagnetic or active implants unsuitable for 7 T. Ten patients (50%) received gross tumor resection before imaging and (C)RT. Eleven patients (55%) presented with newly diagnosed GBM and received CRT according to Stupp et al. (2005) or CRT modified to elderly age and decreased clinical performance according to Perry et al. (2017). Moreover, nine patients (45%) presented with recurrent GBM and received particle RT with or without systemic treatment. Detailed patient characteristics are shown in Table 1.

**TABLE 1:** Patient characteristics (*N* = 20).

	**All patients (*N* = 20)**	**Initial progression (*N* = 9)**	**Initial stable disease (*N* = 7)**	
**Age (years)**				
Median (IQR)	56.5 (47.8–61.2)	61 (58–68)	53 (45.5–60.5)	*p* = 0.2
**Sex**				
Male	11 (55%)	5	4	*p* = 1.0
Female	9 (45%)	4	3	
**KPI (%)**				
Median (IQR)	80 (70–90)	70 (70–92.5)	90 (85–90)	*p* = 0.22
**IDH Status**				
Wild type	18 (90%)	8	6	*p* = 0.47
Mutated	1 (5%)	0	1	
Unknown	1 (5%)	1		
**MGMT methylation**				
Not methylated	4 (20%)	1	2	*p* = 1.0
Methylated	10 (50%)	5	4	
Unknown	6 (30%)	3	1	
**Recurrent disease**				
Yes	9 (45%)	5	3	*p* = 1.0
No	11 (55%)	4	4	
**Gross tumor resection**				
Yes	10 (50%)	3	5	*p* = 0.31
No	10 (50%)	6	2	
**RT**				
60/2 Gy	9 (45%)	2	4	*p* = 0.50
40.05 Gy/2.67 Gy	2 (10%)	2	0	
Particle re-RT	9 (45%)	5	3	
**Concurrent chemotherapy**				
Yes	12 (60%)	5	5	*p* = 0.63
No	8 (40%)	4	2	
**Initial response (3T MRI only)**				
Progressive disease	9 (45%)	8	1	***p* = 0.002**
Stable disease	5 (25%)	0	5	
Pseudoprogression	2 (10%)	1	1	
Not available	4	–	–	
**OS (days)**				
Median (IQR)	247.5 (150–389.8)	154 (124–258)	426 (322–576)	***p* = 0.005**
**PFS (days)**				
Median (IQR)	92.5 (77.3–265.5)	78 (59–86)	267 (243–289)	***p* = 0.001**
**TSC**				
Median (IQR)				
TSC t0 [absolute (mM)]	54.1 (46.6–58.1)	54.0 (39.2–58.8)	54.2 (50.1–58.3)	*p* = 0.76
TSC Δ t1–t0 (% of t0)	2.5 (−4.9 to 13.0)	1.1 (−5.8 to 9.6)	3.4 (−2.6 to 13.8)	*N* = 7/7, *p* = 0.62
TSC Δ t2–t0 (% of t0)	10.5 (1.1–22.5)	9.0 (−0.2 to 24.8)	11.9 (5.2–15.5)	*N* = 5/5, *p* = 1.0
TSC Δ t2–t1 (% of t1)	12.1 (3.2–19.2)	19.3 (14.0–24.3)	1.7 (−2.8 to 10.1)	*N* = 5/5, *p* = 0.056

*Initial response to treatment was evaluated according to the response assessment in neuro-oncology working group (RANO) criteria. The tissue sodium concentration (TSC) was measured at three different time steps: t0, pre-treatment; t1, immediately post-treatment; t2, 6 weeks post-treatment. (N, total number; IQR, interquartile range; KPI, Karnofsky Performance Index; IDH, isocitrate-dehydrogenase; MGMT, O^6^-methylguanine DNA methyltransferase; RT, radiotherapy; Gy, Gray; TMZ, Temozolomide; RBE, relative biological effectiveness; PD, progressive disease; SD, stable disease; PsPD, pseudoprogression; NA, not available; OS, overall survival; PFS, progression-free survival; TSC, tissue sodium concentration; Mm, mmol/l; Δ, difference).*

*Bold indicates statistically significant values with p < 0.05.*

### ^23^Na Magnetic Resonance Imaging at 7T

All ^23^Na images were acquired on a 7T research scanner (Siemens Healthcare, Erlangen, Germany) with a double-resonant (^1^H/^23^Na) quadrature birdcage coil (RAPID Biomedical, Rimpar, Germany). The ^23^Na data was obtained with a density-adapted 3D radial pulse sequence (Nagel et al., 2009) which yielded a nominal resolution of (Δx)^3^ = (3 mm)^3^ (TR/TE = 160 ms/0.35 ms, readout time = 10 ms, number of projections = 4,000, acquisition time = 10:40 min). TE was measured from the center of the RF pulse (shape: rectangular, duration: 600μs) to the start of the readout. Image reconstruction employed an iterative 3D Dictionary Learning Compressed Sensing algorithm (3D-DLCS) (Behl et al., 2016) (block size B = 3 × 3 × 3; dictionary size D = 80; number of samples = 500,000; regularization weighting factor μ = 0.5).

The tissue sodium concentration (TSC) was calculated using two reference vials (0.3–0.6% NaCl). Both B_1_^+^ and B_1_^–^ corrections were applied to reduce transmit and receive inhomogeneities using the double angle method for the field estimations (Insko and Bolinger, 1993).

### Clinical 3T Magnetic Resonance Imaging

All patients underwent clinical MRI at a field strength of 3T before initiation of (C)RT. Furthermore, clinical follow-up MRIs were performed approximately 4 and 12 weeks post-therapy. Routinely, clinical 3T MRI encompassed a pre- and post-contrast T1-weighted sequence (Gadolinium based contrast agent; TE = 4.04 ms, TR = 1,710 ms, FoV = 256 × 256 mm^2^, matrix = 512 × 512, slice thickness = 1 mm) and a fluid-attenuated inversion recovery (FLAIR) sequence (TE = 135 ms, TR = 8,500 ms, FoV = 230 × 172 mm^2^, matrix = 256 × 192, slice thickness = 5 mm) used for response assessment in accordance with the updated RANO criteria (Wen et al., 2010).

### Image Processing

The clinical 3T MRI from the pre-therapy examination and the clinical 3T MRI from the 4-week post-therapy follow-up were co-registered to the radiotherapy planning CT scan by an automatic multi-modal rigid algorithm in MITK (Nolden et al., 2013). Subsequently, an experienced radiologist (DP, 8 years of experience) segmented the Gadolinium contrast enhancing (gdce) areas on T1-weighted 3T MRI together with hyperintense areas on FLAIR T2-weighted 3T MRI on pre- and post-therapy imaging. Necrosis was excluded carefully from the segmentation, resulting in the whole tumor area excluding necrosis. Finally, the segmentation derived from pre-therapy 3T MRI was co-registered to the initial (t_0_) 7T ^23^Na MRI, while the segmentation derived from the 4-week post-therapy 3T MRI was co-registered to 7T ^23^Na MRI at both t_1_ and t_2_ to extract TSC values from the tumor region. An example of the tumor contouring and image registration procedures can be found in Supplementary Figure 1.

### Response Assessment

Patients were routinely scheduled for clinical follow-up visits together with the 3T MRI examinations approximately 4 and 12 weeks post-therapy, as described above. These follow-up visits included thorough clinical examination as well as overall assessment of the radiological findings together with clinical information by a neurooncologist.

Study participants were classified as complete response (CR), partial response (PR), stable disease (SD), or progressive disease (PD) based on radiographic findings and clinical information by the department of neuroradiology and neuro-oncology in accordance with the updated RANO criteria (Wen et al., 2010). To account for possible cases of PsPD, we followed the recommendations of the updated RANO criteria: Each PD in the first follow-up needed to be confirmed in the second follow-up or via biopsy (Wen et al., 2010). Performance of a biopsy was indicated clinically. If the second follow-up or biopsy did not confirm a PD, PsPD was stated. For further analysis, we additionally conducted a radiographic response assessment on clinical 3T MRI only with regard to signal changes (T1 pre- and post-contrast, T2-FLAIR) without using information on clinical status or histopathology (biopsy). A missed follow-up examination due to death or massive deterioration was counted as PD in both assessments.

PFS was calculated from the date of the baseline 7T ^23^Na MRI (t_0_) to the date of first progression or death from any cause. OS was calculated from the date of the baseline 7T ^23^Na MRI (t_0_) until death from any cause. At the time of statistical analysis, all patients had reached both endpoints.

### Statistics

The median TSC inside the whole tumor area excluding necrosis was calculated at t_0_, t_1_, and t_2_. Subsequently, the differences in median TSC between all-time steps were calculated. Those differences were normalized to the TSC of the earlier time step, yielding the normalized TSC changes. Clinical parameters, baseline median TSC at t_0_ and the normalized TSC changes were compared between patients with initial SD and patients with initial PD using Fisher’s exact test for non-continuous variables and Mann-Whitney *U*-tests for continuous variables. Furthermore, univariate proportional hazard models of PFS and OS were developed for different clinical and imaging parameters with the following predictor variables: patient age, sex, Karnofsky Performance Index (KPI), tumor recurrence (binary: recurrent, newly diagnosed), O^6^-methylguanine DNA methyltransferase (MGMT) methylation (binary: yes, no), gross surgical resection (binary: yes, no), type of CRT (binary: standard of care, other), initial response according to RANO or radiographic findings only (binary: PD, SD), the baseline TSC at t_0_ and the normalized TSC changes between all-time steps. The according hazard ratios (HR) and standard errors (SE) were calculated as maximum-likelihood estimates (MLE) and *p*-values were derived from the likelihood ratio test. In the proportional hazard model for PFS with RANO response status as predictor variable, MLEs for HRs did not converge and thus failed to yield a result. Hence, the corresponding univariate proportional hazard models employed Firth’s penalized likelihood method and the computation of profile likelihood confidence intervals to yield valid estimates and *p*-values (Heinze and Ploner, 2002). All statistical evaluation employed R version 4.0.3.

### Ethics

According to the declaration of Helsinki, this study received approval by the local ethics board (IRB number: S343/2016) and 7T MRI examinations were only initiated after a written informed consent was obtained from the patient.

## Results

According to the RANO criteria, the course of nine patients was classified as initial PD, whereas seven patients presented with initial SD, two of which with PsPD. One patient received post-treatment biopsy, which confirmed a PsPD. Four patients could not be classified due to cancelation of treatment or missing clinical follow-up. Classification according to changes on clinical 3T MRI only yielded similar results with two disagreements, thus showing a statistically significant association with the original RANO assessment (*p* = 0.002, Table 1).

Patients with initial PD showed a tendency toward higher age, lower KPI and fewer gross tumor resections, but no statistically significant differences were found between the initial PD and initial SD group concerning clinical baseline parameters. However, there was a statistically significant difference in PFS [Median PFS (IQR) (days): *PD* = 78 (59–86), *SD* = 267 (243–289), *p* = 0.001] and OS [Median OS (IQR) (days): *PD* = 154 (124–258), *SD* = 426 (322–576), *p* = 0.005] between the two groups (Table 1).

**FIGURE 1:**
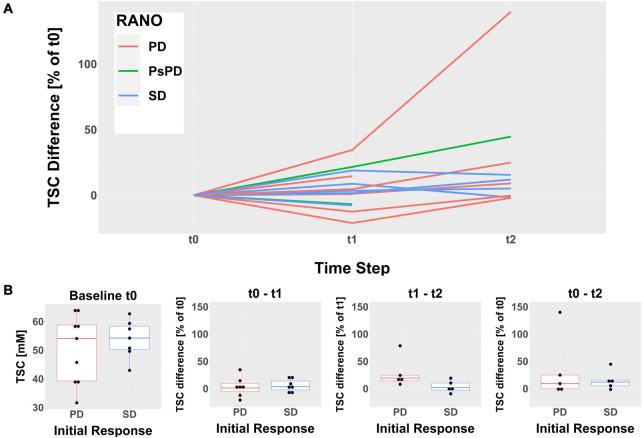
Evolution of the median tissue sodium concentration (TSC) inside the whole tumor areas excluding necrosis following treatment. The time steps refer to the pre-therapy imaging (t_0_), the follow-up immediately post-therapy (t_1_) and the follow-up 6 weeks post-therapy (t_2_). **(A)** The descriptive analysis shows a heterogeneous evolution of TSC for all patients that does not evidently reflect the RANO response evaluation. The differences between t_0_ and t_1_ (at time step t_1_) as well as between t_0_ and t_2_ (at time step t_2_) were normalized to the baseline TSC at t_0_. **(B)** Boxplots showing the median TSC at t_0_ (left) as well as the normalized TSC differences between all-time steps grouped by initial response according to RANO. None of the observed intergroup differences reached statistical significance.

**FIGURE 2:**
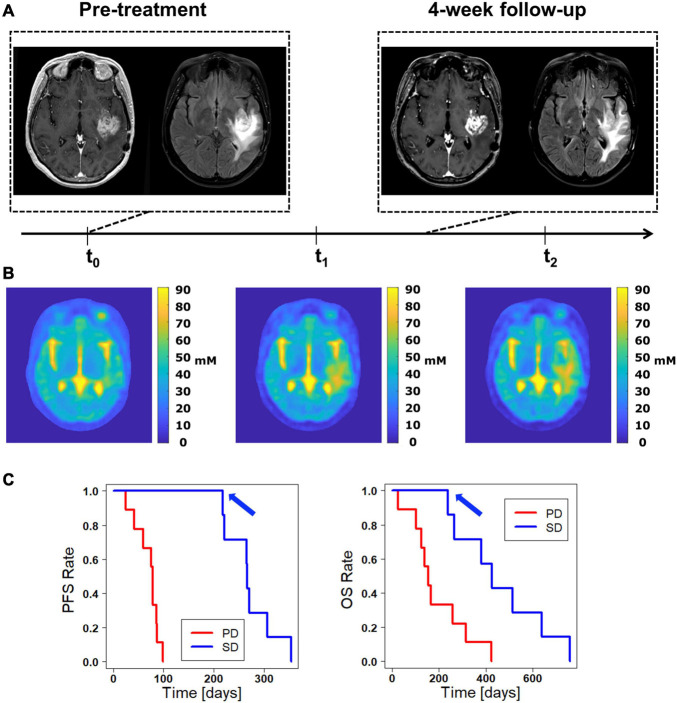
Discrepancy between clinical 3T MRI and 7T ^23^Na MRI. **(A)** The clinical 3T MRI of this patient presents only minor changes when comparing the pre-therapy baseline with the 4-week follow up, suggesting an initial stable disease (SD) according to the RANO criteria. **(B)** The tissue sodium concentration (TSC) shows a successive signal increase both inside the contrast enhancing region and within peritumoral edema. One could deduce a progressive disease (PD) with additional tumor infiltration and consecutively increased signal. On the other hand, a treatment response with successive tumor necrosis and reactive perifocal edema would also be a possible explanation. **(C)** The Kaplan-Meier curves for progression free survival (PFS, left) and overall survival (OS, right) show the significant prediction of PFS and OS by the RANO classification found in this study. Accordingly, the patient shown in A and B, which is rated as initial SD, lies on the favorable upper curve (blue arrow).

Descriptive analysis of TSC inside the whole tumor region excluding necrosis following treatment revealed a heterogeneous development over all patients (Figure 1A). The pre-therapy (t_0_) median TSC did not differ significantly between cases of initial SD and initial PD according to RANO [Median TSC (IQR) (mM): *PD* = 54.04 (39.22–58.77), *SD* = 54.18 (50.14–58.34), *p* = 0.76]. Similarly, the normalized TSC changes did not show a statistically significant difference between cases of initial SD and initial PD according to RANO, but there was a tendency toward a stronger increase in TSC from t_1_ to t_2_ in early progressing tumors [Normalized ΔTSC_*t*__0__–__*t*__1_ (IQR): *PD* = 1.07% (−5.84 to 9.58%), *SD* = 3.42% (−2.62 to 13.8%), *p* = 0.62; normalized ΔTSC_*t*__0__–__*t*__2_ (IQR): *PD* = 8.99% (−0.24 to 24.84%), *SD* = 11.92% (5.15–15.49%), *p* = 1; normalized ΔTSC_*t*__1__–__*t*__2_ (IQR): *PD* = 19.27% (14.03–24.33%), *SD* = 1.68% (−2.83 to 10.11%), *p* = 0.056]. Similar results were obtained when analyzing uncorrected, absolute TSC values and differences (Supplementary Figure 2 and Supplementary Table 1). Table 1 and Figure 1B yield further details on the development of TSC following CRT for cases of initial SD and initial PD according to RANO. The discrepancy between findings on clinical 3T MRI and development of TSC is illustrated qualitatively for one patient case in Figure 2.

Univariate Cox proportional hazard models revealed that both the RANO criteria assessment of initial response as well as the 3T MRI only assessment of initial response yielded statistically significant predictors of PFS [RANO: HR (95% CI) = 0.02 (0.0001–0.21), *p* = 7⋅10^–5^; 3T MRI only: HR (95% CI) = 0.1 (0.02–0.49), *p* = 0.0009] and OS [RANO: HR (95% CI) = 0.17 (0.04–0.65), *p* = 0.005; 3T MRI only: HR (95% CI) = 0.26 (0.08–0.87), *p* = 0.02]. Furthermore, standard of care CRT was associated with significantly improved PFS [HR (95% CI) = 0.312 (0.1–1.0), *p* = 0.046]. Conversely, none of the TSC measures yielded a significant prediction of PFS or OS. Table 2 provides detailed statistical findings of the survival analysis.

**TABLE 2:** Results of the statistical analysis: association with PFS and survival.

**Univariate proportional hazard model**						
**Variable**	**Progression free survival**			**Overall survival**		
	**N**	**HR (95% CI)**	***p*-value**	**N**	**HR (95% CI)**	***p*-value**

Age	16	1.1 (1–1.1)	0.054	20	1 (0.99–1.1)	0.13
Sex	16	0.58 (0.19–1.8)	0.34	20	0.77 (0.29–2.1)	0.6
KPI	16	0.96 (0.92–1)	0.11	19	0.98 (0.94–1)	0.19
MGMT status	12	0.96 (0.25–3.7)	0.95	14	0.99 (0.3–3.3)	0.98
Recurrence	16	1.7 (0.57–5.1)	0.33	20	1.6 (0.65–4.2)	0.3
Resection	16	2.1 (0.71–6.0)	0.18	20	2.4 (0.94–6.2)	0.07
**CRT**	**16**	**0.31 (0.1**–**1)**	**0.046**	20	0.41 (0.16–1.1)	0.07
**Response (RANO)**	**16**	**0.02 (10**^–^**^4^**−0.21)*	**6.6⋅10** ^–^ ^5*^	**16**	**0.17 (0.04**–**0.65)**	**0.005**
**Response (3T MRI w/o clinical data)**	**16**	**0.1 (0.02**–**0.49)**	**0.0009**	**16**	**0.26 (0.08**–**0.87)**	**0.02**
TSC Median t0	16	0.98 (0.92–1.1)	0.62	20	1 (0.95–1.1)	0.85
TSC Δ t1–t0	14	0.97 (0.92–1)	0.27	14	0.97 (0.93–1)	0.24
TSC Δ t2–t0	10	1 (0.98–1)	0.83	10	0.99 (0.98–1)	0.51
TSC Δ t2–t1	10	1 (0.99–1.1)	0.25	10	1 (0.97–1)	0.96

*N, number of patients; HR, hazard ratio; CI, confidence interval; KPI, Karnofsky Performance Index; MGMT, O^6^-methylguanine DNA methyltransferase; CRT, chemoradiotherapy; TSC, tissue sodium concentration; Δ, relative TSC difference; t0, pre-therapy ^23^Na MRI; t1, ^23^Na MRI immediately post-therapy; t2, ^23^Na MRI 6-weeks post-therapy.*

*The corresponding analysis was based on Firth’s penalized likelihood method with the computation of profile likelihood confidence intervals and p-values according to penalized likelihood ratio tests.

*Bold indicates statistically significant values with p < 0.05.*

Since the evolution of TSC after treatment might have been influenced by prior treatments, we also conducted a descriptive analysis of TSC in newly diagnosed and recurrent tumors separately. This small subgroup analysis did not reveal clear differences between newly diagnosed and recurrent tumors (Supplementary Figure 3).

## Discussion

To our knowledge, this is the first prospective investigation of ^23^Na MRI at an ultra-high field strength of 7 T to evaluate treatment response in GBM patients. Our data demonstrates heterogeneous changes of TSC following CRT, which suggests that ^23^Na MRI is sensitive to treatment-related effects. The TSC changes did not correlate with standard of care response assessment or clinical 3T MRI findings in general, so that ^23^Na MRI might offer complementary biological information. However, the evaluation of TSC in the tumor did not enable a prediction of tumor progression or patient survival. Therefore, future investigations need to elucidate the reasons for treatment-related changes in TSC and their clinical value.

### ^23^Na-Imaging and Origins of Altered Tissue Sodium Concentration

Compared to conventional proton MRI, ^23^Na MRI is limited by a reduced SNR due to the approx. 10.000-fold lower concentration of ^23^Na ions than protons in the human body (Shah et al., 2016; Regnery and Platt, 2021). SNR increases with increasing magnetic field strength, so that UHF-MRI scanners > 3T can significantly improve image quality in ^23^Na MRI (Konstandin and Schad, 2014; Shah et al., 2016). Therefore, we employed an ultra-high field strength of 7T to optimize the SNR. Currently, more and more centers worldwide are acquiring UHF MRI scanners, which are likely to become increasingly applied in neuro-oncology, particularly to support ^23^Na-imaging, in the near future (Platt et al., 2021).

TSC depends on intra- and extracellular volumes with the underlying ^23^Na ion distribution (Ouwerkerk et al., 2003; Thulborn et al., 2009). Therefore, the significant elevation of TSC inside of gliomas (Turski et al., 1987; Ouwerkerk et al., 2003; Regnery et al., 2020) might be explained due to both higher intracellular ^23^Na content of malignant cells (Cameron et al., 1980; Rotin et al., 1989) and enlarged extracellular space in gliomas (Bakay, 1970; Bruehlmeier et al., 2003; Zamecnik et al., 2004). The contribution of either compartment to the increased signal remains controversial (Ouwerkerk et al., 2003; Thulborn, 2016). Furthermore, it needs to be acknowledged that brain tumors can be accompanied by non-infiltrative peritumoral edema, blood brain barrier disruption and different tumor vascular structure, all of which might lead to an increased TSC as well (Turski et al., 1986, 1987; Driver et al., 2020).

### Tissue Sodium Concentration as Response Predictor

Concerning treatment-related changes in TSC, previous findings from animal GBM models suggested that tumor TSC rises few days after an effective chemotherapy, which could reflect intracellular sodium overload or cellular shrinkage due to apoptosis and necrosis (Schepkin et al., 2005, 2006). Those preclinical studies investigated short time tumor response to few doses of chemotherapy without incorporating RT or a treatment protocol over several weeks. Nevertheless, similar tendencies were suggested by preliminary reports of clinical observations during radiotherapy (Thulborn et al., 2009). Moreover, several case reports revealed ^23^Na signal changes inside gliomas following CRT which were different from findings in clinical imaging, but the underlying biochemical reasons remained undiscovered (Thulborn et al., 2009; Laymon et al., 2012; Haneder et al., 2015). Lastly, a recent clinical study of ^23^Na MRI at 3T during concomitant CRT in GBM patients hypothesized that TSC may be used to measure tumor cell kill by longitudinal measurements of the brain volume with elevated TSC, representing the residual tumor volume, and its nominal TSC, which may be used to estimate the cell volume fraction (Thulborn et al., 2019).

This study found dichotomous changes of TSC-derived tumor volumes and suggested TSC as measure of tumor cell kill (Thulborn et al., 2019). However, a significant correlation between TSC and PFS was not found, which led the authors to conclude that tumor cell kill due to concomitant CRT alone might have a minor impact on survival when compared to the additional effect of adjuvant chemotherapy (Thulborn et al., 2019).

Our descriptive analysis revealed heterogeneous changes of TSC following (C)RT, which supports the idea that TSC quantifies different responses to treatment. Moreover, there was a trend toward a stronger TSC increase from t_1_ to t_2_ in early progressing tumors according to RANO criteria. Nevertheless, we did not find a significant correlation of the development of TSC with the clinical response assessments based on the RANO criteria. These findings agree with the previous reports which suggest that TSC generates information different from established clinical imaging (Laymon et al., 2012; Haneder et al., 2015; Thulborn et al., 2019). However, just as reported by the recent trial at 3T (Thulborn et al., 2019), TSC did not show a significant correlation with PFS or OS either. Our methods were complementary to this previous trial in many regards. TSC was measured at 7T, which provides a higher signal-to-noise-ratio (SNR). In addition, TSC was measured immediately after therapy (t_1_) as well as 6-weeks post-therapy (t_2_), therefore including later timepoints. Furthermore, our investigation encompassed the additional correlation of clinical classifications of response to therapy with PFS and OS, which yielded significant results.

Hence, TSC seems to reflect treatment effects which are not visible on clinical MRI, but a straightforward correlation with tumor cell kill has not been demonstrated yet. There was a trend toward a stronger TSC increase from t_1_ to t_2_ in early progressing GBM that could reach statistical significance in larger patient cohorts. However, TSC did not correlate with PFS and OS while clinical response correlated well with PFS and OS. Presumably, various factors besides mere tumor cell kill could have an overlapping influence on TSC, namely therapy-related non-infiltrative edema (Turski et al., 1986, 1987; Hashimoto et al., 1991; Ouwerkerk et al., 2003) and blood-brain-barrier disruption (Turski et al., 1986). Effective tumor cell kill may reduce blood brain barrier disruptions and brain edema, with a consecutive decrease of TSC in the corresponding regions (Thulborn et al., 2009). On the other hand, CRT itself can also cause blood brain barrier disruptions and brain edema, leading to the well-known pseudoprogressions with potential elevations of the TSC. Therefore, future approaches should aim to combine ^23^Na MRI with proton MRI (e.g., diffusion-weighted imaging, perfusion-weighted imaging) to better understand different treatment effects besides tumor cell kill. Since ^23^Na MRI offers information that is technically and biologically independent from established proton MRI applications, it might be highly useful in future multiparametric, machine-learning based approaches.

### Limitations and Strengths

Several limitations of this study need to be acknowledged. Firstly, the patient cohort is relatively small and heterogeneous. Most patients had a comparatively poor prognosis due to a high number of relapsing tumors and only 50% gross tumor resections. This limits the power of our analysis. In addition, the use of different RT regimes could have led to different treatment reactions in normal tissues, e.g., different grades of blood brain barrier disruption. In particular, patients receiving (re-) radiotherapy of a relapsing GBM tend to experience pseudoprogressions and radionecrosis more often, and such treatment-related changes can confound the TSC. Nevertheless, all patients received RT as main treatment and the aim was to predict therapy response and survival, which should be possible if ^23^Na MRI yields a straightforward measure of tumor cell kill. Clinical response assessments yielded a significant correlation with PFS and OS, whereas the TSC quantification in our setup failed to do so. However, it cannot be excluded that at higher SNR or higher spatial resolutions (e.g., due to B_0_ > 7T, longer measurement times, use of array receiver coils) a correlation might become observable. Secondly, clinical 3T MRI and 7T ^23^Na MRI were not performed at completely parallel follow-ups, which could have impeded the coregistration of the segmentations. In the past, different approaches have been used to analyze ^23^Na MR images, mostly relying on segmentations defined on clinical MRI (Turski et al., 1986; Heinze and Ploner, 2002; Thulborn, 2016; Driver et al., 2020). A recent study investigating ^23^Na MRI at 3T for response assessment in GBM patients used a semi-automatic approach to delineate tumor volumes directly on ^23^Na images based on TSC cut-offs (which were created using clinical MRI) (Thulborn et al., 2019). This approach did not prove feasible in our data because of elevated TSC around CSF-spaces due to partial volume effects despite a high SNR at the 7T field strength. Lastly, the clinical assessment of treatment response might still be constrained by misclassifications due to pseudoprogression despite following the RANO recommendations as current golden standard.

The strengths of this study include the prospective design and the use of a 7T ultra-high field scanner which offers high SNR compared to clinical field strengths. As opposed to previous studies that analyzed TSC changes over a short period of time, this study encompassed longitudinal TSC measurement up to 6 weeks after treatment. Furthermore, correlation with several clinical and radiographic parameters was performed to compare the predictive capacity of ^23^Na MRI with clinical standard information.

## Data Availability Statement

The raw data supporting the conclusions of this article are available only upon scientific request because patient data are included. The corresponding author (DP) may be contacted to request the data.

## Ethics Statement

The studies involving human participants were reviewed and approved by the University Heidelberg Institutional Review Board Alte Glockengießerei 11/1, 69115 Heidelberg, Germany. The patients/participants provided their written informed consent to participate in this study.

## Author Contributions

DP, SA, SRe, and NB: study conceptualization. NB and TP: technical methodology. DP, SRe, NB, and KD-H: examinations. SRe, NB, TP, NW, PW, and LK: data processing. SRe and DP: statistical analysis and writing first draft. SA, JD, H-PS, ML, SRi, WW, and MB: scientific supervision. All authors reviewing and editing first draft.

## Conflict of Interest

NB was employed by the company Siemens Healthcare GmbH. The remaining authors declare that the research was conducted in the absence of any commercial or financial relationships that could be construed as a potential conflict of interest.

## Publisher’s Note

All claims expressed in this article are solely those of the authors and do not necessarily represent those of their affiliated organizations, or those of the publisher, the editors and the reviewers. Any product that may be evaluated in this article, or claim that may be made by its manufacturer, is not guaranteed or endorsed by the publisher.
