# Supplementary material for: Assessment of Sodium MRI at 7 Tesla as Predictor of Therapy Response and Survival in Glioblastoma Patients
Source: Front Neurosci. 2021 Dec 1;15:782516. doi: 10.3389/fnins.2021.782516 (PMC8671745; doi:10.3389/fnins.2021.782516)

Supplementary Figure 1: Contouring of tumor subregions and registration. A) Raw images. B) Overlay of segmentations: edema (yellow), Gadolinium contrast enhancement (red) and necrosis (purple). T1w: T1-weighted imaging with Gadolinium contrast agent. FLAIR: Fluid-attenuated inversion recovery. ^{23}Na: sodium imaging (raw data, without interpolations performed).

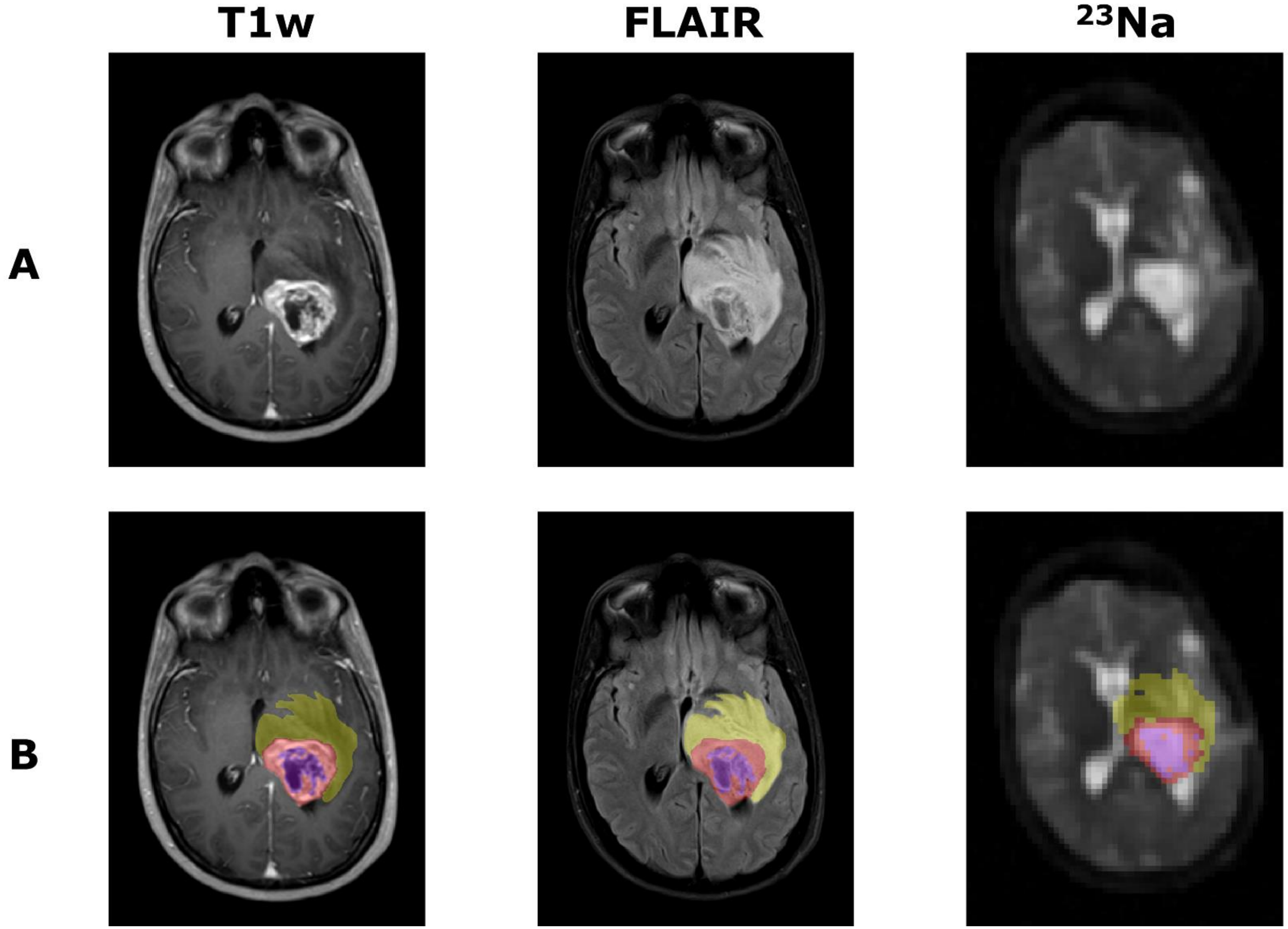

Supplementary Figure 2: Evolution of the absolute tissue sodium concentration (TSC) values. The time steps refer to the pre-therapy imaging (t_0), the follow-up immediately post-therapy (t_1) and the follow-up 6 weeks post-therapy (t_2). Baseline values and differences are both plotted as absolute values.

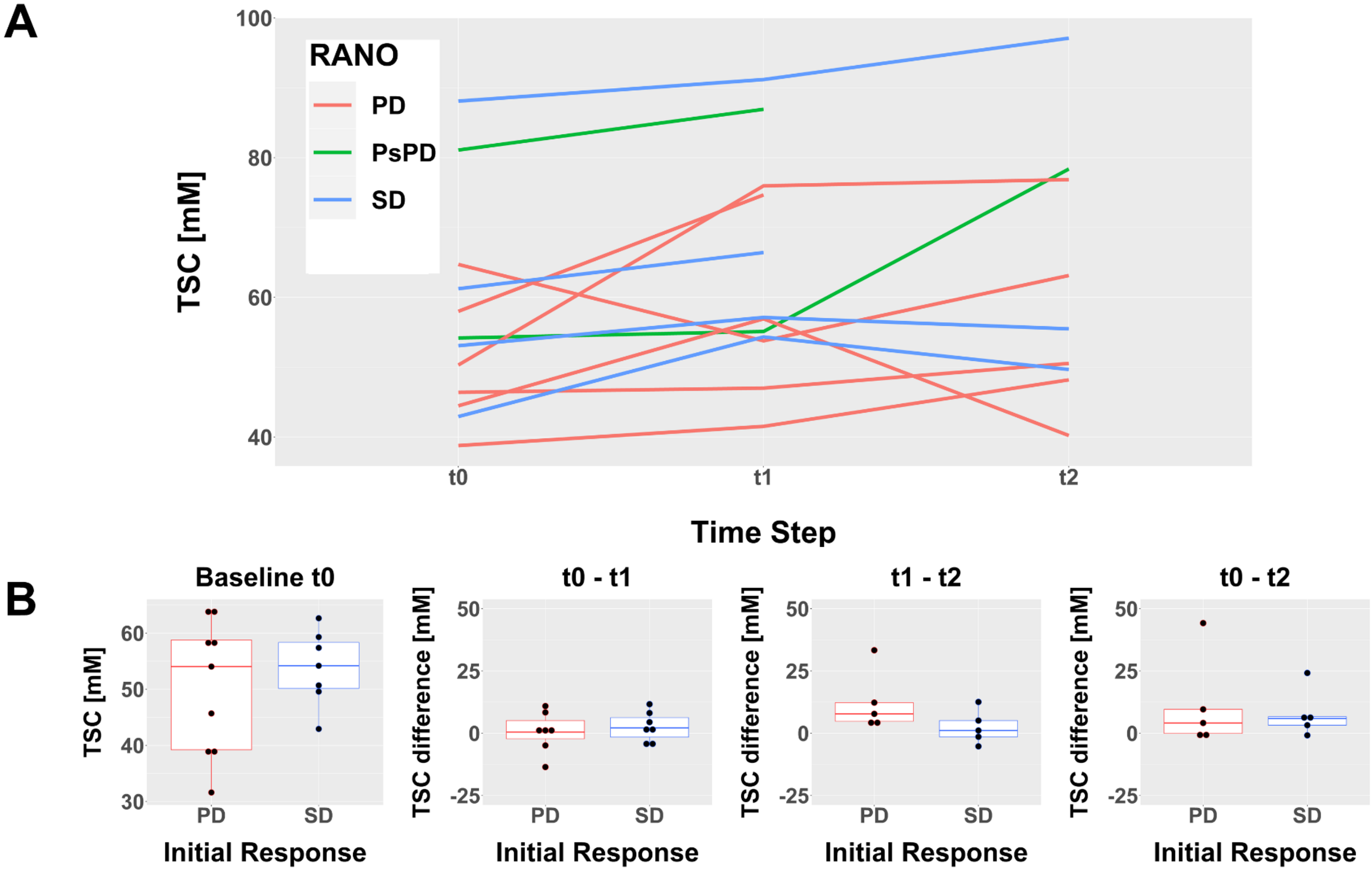

Supplementary Table 1: Absolute tissue sodium concentration (TSC) values and differences between time points.

	**All Patients (N = 20)**	**Initial Progression (N = 9)**	**Initial Stable Disease (N = 7)**	
TSC t0 [mM]	54.1 (46.6 - 58.1)	54.0 (39.2 - 58.8)	54.2 (50.1 - 58.3)	p = 0.76
TSC Δ t1 – t0 [mM]	1.3 (-2.9 – 7.2)	0.5 (-2.2 – 5.1)	2.1 (-1.6 – 6.3)	N= 7 / 7, p = 0.54
TSC Δ t2 – t0 [mM]	5.0 (0.7 – 8.9)	4.1 (-0.1 – 9.6)	5.9 (3.2 – 6.7)	N= 5 / 5, p = 1.0
TSC Δ t2 – t1 [mM]	5.0 (1.7 - 11.2)	7.8 (4.8 - 12.3)	1.1 (-1.4 - 5.1)	N= 5 / 5, p = 0.22

Values are given as median (interquartile range). N: patient number.

Supplementary Figure 3: Evolution of the tissue sodium concentration (TSC) in cases of newly diagnosed and recurrent tumors. The time steps refer to the pre-therapy imaging (t_0), the follow-up immediately post-therapy (t_1) and the follow-up 6 weeks post-therapy (t_2).

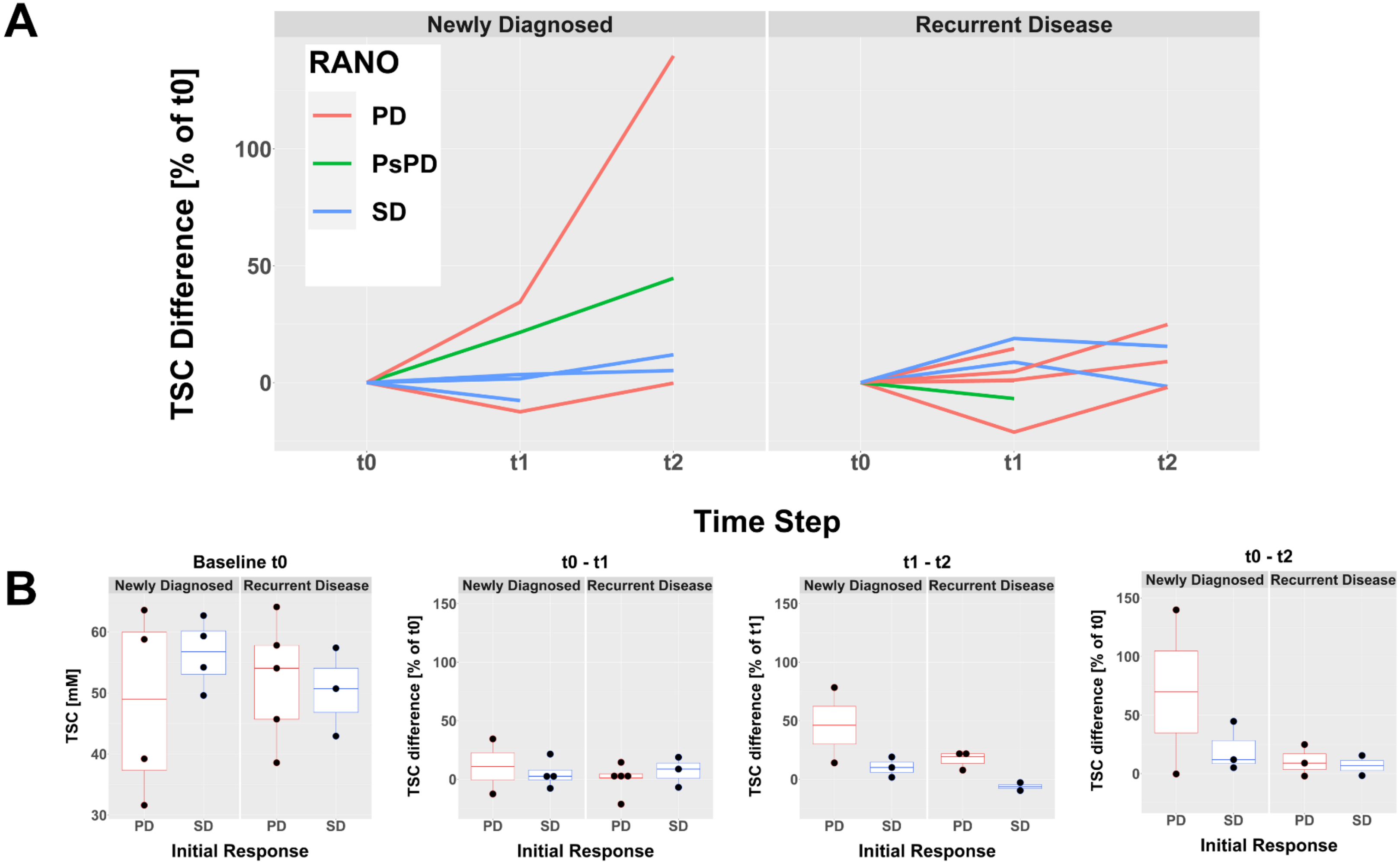

Supplement: Supplementary file 1 [file Data_Sheet_1.PDF]
